# Society Meetings

**Published:** 1905-02

**Authors:** 


### SOCIETY MEETINGS.

The Medical Society of the State of New York is holding its ninety-ninth annual meeting at Albany, just as these pages reach the eyes of our Buffalo readers. The President is Dr. Hamilton D. Wey, of Elmira, and the secretary is Dr. Frederic C. Curtis, of Albany. The dinner to the ex-presidents was given at the

Fort Orange Club Monday evening, January 30, by Dr. W. G. Macdonald, of Albany. The society convened Tuesday morning, January 31, at 9.30. Tuesday evening at 8.30 the session was held in the senate chamber at which an address was given by Dr. Charles Harrington of Boston, and the president's annual address was delivered. At 9.30 a reception to Dr. L. S. McMurtry, President of the American Medical Association, was given by Dr. Leo Händel Neuman, of Albany. The annual dinner was given at the Ten Eyck, Wednesday evening at 8.30, immediately after the president's reception. At 12 o'clock noon Thursday, February 2, a special meeting was held to take further action on the unification of the medical profession in the empire state. A large number of important scientific contributions were read and discussed.

---

At the annual meeting of the Southern Surgical and Gynecological Association, held at Birmingham, Ala., December 13-15, 1904, the following-named officers were elected: president, Lewis C. Bosher, Richmond; first vice-president, John D. S. Davis, Birmingham; second vice-president, I. S. Stone, Washington; secretary, W. D. Haggard, Nashville; treasurer, Charles M. Rosser, Dallas. The next meeting will be held at Louisville, in December, 1905.

The monument erected by the association to its founder, the late Dr. W. E. B. Davis, was unveiled in Capitol Park, Wednesday, December 14, at 11 o'clock, in the presence of 5,000 people, including the members of the association. An invocation by Rev. Dr. L. S. Handley, an address by Dr. Charles M. Rosser, of Dallas, Texas, the unveiling by Elizabeth and Margaret Davis, the little daughters of Dr. Davis, an address by Dr. R. M. Cunningham, acting Governor of the State of Alabama, an address by Hon. John C. Forney, representative of Mayor Drennan, who was unavoidably absent, constituted the ceremonies.

---

The Canandaigua Medical Society held its fortieth anniversary meeting January 19, 1905, at the residence of Dr. Matthew R. Carson. But two of the original members survive, Dr. John B. Chapin, of Philadelphia, and Dr. Carson himself.

---

At the annual meeting of the Medical Society of the County of Erie held January 10, 1905, the following-named officers were elected: president, John D. Macpherson, Akron; vice-president, A. H. Briggs, Buffalo; secretary, F. C. Gram, Buffalo; treasurer, D. C. Greene, Buffalo. Censors: H. R. Hopkins, J. H. Grant, Irving W. Potter, Ernest Wende and F. E. Fronczak, Buffalo. The full proceedings will be published in a forthcoming number of the JOURNAL.

---

THE Esculapian Club held its regular monthly meeting at the Hotel Touraine, Thursday evening, January 19, 1905, at which it was entertained by Dr. C. J. Reynolds. The subject discussed was The insanity of pubescence, by G. R. Trowbridge.

---

THE Elmira Academy of Medicine held its annual meeting Wednesday evening, January 4, 1905. Program: Plastic surgery—is it a lost art? The repair of some of the common accidents incident to parturition, early and late, Joseph Price, Philadelphia; Perineal prostatectomy, William B. Jones, Rochester; President's address, Hamilton D. Wey.

---

THE Buffalo Academy of Medicine held meetings during the months of December and January as follows:

*Section on Obstetrics and Gynecology.*—Tuesday evening, December 27. Program: Experiences in the prechloroform era, Silas Hubbard, "42"; Autointoxication and its relation to the pelvic organs, J. E. Walker, Hornellsville, N. Y.

*Section on Surgery.*—Tuesday evening, January 3. Program: (*a*) The treatment of chronic laryngeal stenosis, John Rogers, New York; (*b*) The treatment of chancre and chancroid, David E. Wheeler; (*c*) Protozoa in the blood of frogs, Mr. Joseph Lewis.

*Section on Medicine.*—Tuesday evening, January 10. Program: (*a*) The value of certain procedures in the treatment of pneumonia, DeLancey Rochester; (*b*) Some remarks on the action of the Röntgen rays, Grover W. Wende.

*Section on Pathology.*—Thursday evening, January 19. Program: A preliminary report on an experimental research concerning the nature of immunity in cancer, H. R. Gaylord, G. H. A. Clowes, Mr. F. W. Baeslock.

*Section on Obstetrics and Gynecology.*—Tuesday evening, January 24. Program: The nature and surgical treatment of chronic facial and obstetrical palsies, L. Pierce Clark, A. J. Taylor, New York.

